# Osteoprotective effects of *Fructus Ligustri Lucidi *aqueous extract in aged ovariectomized rats

**DOI:** 10.1186/1749-8546-5-39

**Published:** 2010-11-29

**Authors:** Chun Hay Ko, Wing Sum Siu, Ching Po Lau, Clara Bik San Lau, Kwok Pui Fung, Ping Chung Leung

**Affiliations:** 1 Institute of Chinese Medicine, The Chinese University of Hong Kong, Shatin, Hong Kong, China; 2 School of Biomedical Sciences, The Chinese University of Hong Kong, Shatin, Hong Kong, China

## Abstract

**Background:**

*Fructus Ligustri Lucidi *(FLL) is a commonly used herb for treating bone disorders in Chinese medicine. The present study investigates the anti-osteoporotic activity of FLL aqueous extract in the model of postmenopausal bone loss in aged ovariectomized (OVX) female rats.

**Methods:**

After eight weeks of treatment of FLL or water, the lumbar spine was scanned by peripheral quantitative computed tomography (pQCT). Effects of FLL water extract on osteogenic and adipogenic differentiations in rat mesenchymal stem cells (MSCs) were assessed by biochemical methods and staining.

**Results:**

FLL aqueous extract significantly inhibited bone mineral density (BMD) loss in total, trabecular and cortical bones without affecting body weight and uterus wet weight. FLL extract significantly promoted osteogenesis and suppressed adipogenesis in MSCs as indicated by the elevated alkaline phosphatase activity, calcium deposition levels and decreased adipocyte number in a dose-dependent manner without cytotoxic effects. Real-time PCR analysis revealed significant increase of osteoprotegerin (OPG)-to-receptor activator for nuclear factor-κB ligand (RANKL) mRNA, indicating a decrease in osteoclastogenesis.

**Conclusion:**

The present study demonstrates the osteoprotective effects of FLL aqueous extract on aged OVX rats, stimulation of osteogenesis, inhibition of adipogenesis and osteoclastogenesis in MSCs.

## Background

*Fructus Ligustri Lucidi *(*Nuzhenzi*, FLL), the fruit of *Ligustrum lucidum*, is a major herb for treating aged-related diseases [1]. Our previous study demonstrated that an anti-osteoporosis herbal formula containing *Herba Epimedii*, *Fructus Ligustri Lucidi *and *Fructus Psoraleae *at a ratio of 5:4:1 preserved bone mineral density in osteoporotic rats [2]. FLL ethanol extract modulated the turnover of bone and the calcium balance in ovariectomized rats [3]. FLL ethanol extract improved bone properties in aged ovariectomized rats and enhanced the mineralization process on rat UMR-106 cells [4]. However, little is known about the osteoprotective effects of FLL aqueous extract.

Aged ovariectomized rats and bone marrow mesenchymal stem cells (MSCs) are common animal and cell models used to demonstrate osteoprotective effects. MSCs are pluripotent progenitor cells giving rise to osteoblasts, adipocytes, chondrocytes and myocytes. There is a reciprocal relation between the differentiation of adipocytes and osteoblasts [5]. Clinical studies found an increase in differentiation of MSCs into adipocytes instead of osteoblasts in a variety of osteoporosis [6]. Therefore, the enhancement of osteogenesis with a concomitant decrease in adipogenesis may provide a therapeutic target to the treatment of osteoporosis by increasing bone formation through diverting the adipogenesis in MSCs to osteogenesis [7]. The present study aims to investigate whether FLL aqueous extract (1) prevents the bone mineral loss in lumbar spine; (2) enhances osteogenesis and inhibits adipogenesis in MSCs derived from the rat *in vitro*.

## Methods

### Preparation of FLL aqueous extract

FLL was purchased from a Chinese medicine shop in Hong Kong. The dried fruits were authenticated with high performance liquid chromatography (HPLC) according to the Pharmacopoeia of China [8]. A voucher sample (ICM-200402567) was deposited in the Institute of Chinese Medicine, The Chinese University of Hong Kong. Raw FLL (250 g) was boiled twice with 4L of distilled water for two hours under reflux. The aqueous extract was collected and filtered. The filtrate was then concentrated under reduced pressure at 50°C and lyophilized into powder. The extraction yield was 20% (w/w). The contents of oleanolic acid and ursolic acid (chemical markers) were 0.01% (w/w) and 0.015% (w/w) respectively. The extract powder was stored in desiccators at room temperature before use.

### Animal model and experimental design

Forty (40) 14-month-old female Sprague-Dawley rats were used and housed five per cage in room maintained at 22°C with a 12-hour light-dark cycle. The rats were randomized and equally divided into five groups: (1) Sham: sham-operated group, (2) OVX: ovariectomized group with water administration, (3) OVX+FLL(Low): ovariectomized group treated with 0.35 g/kg/day FLL extract, (4) OVX+FLL(High), ovariectomized group treated with 0.7 g/kg/day FLL extract, (5) OVX+raloxifene: ovariectomized group treated with 2.5 mg/kg/day raloxifene (as positive control). This dosage of raloxifene produced (data not shown) significant protective effects on bone in OVX rats. Seventeen (17) grams of a modified diet containing low mineral content (0.2% calcium and 0.3% phosphorus) were supplied per rat per day after surgery, with water *ad libitum*. The rationale for providing reduced dietary mineral was to mimic the poor intestinal calcium absorption aging condition in aged-postmenopausal women [9]. Three weeks after ovariectomy, FLL aqueous extract was orally administrated to each rat for eight weeks. The two dosages of FLL used in the study was based on the human equivalent doses of the raw herb used according to the Pharmacopoeia of China (6-12 g) with 6 g being the low dose and 12 g the high dose [8]. The dose of FLL used was calculated on the basis of the adult dose used (6 or 12 g raw herb) and the yield of the extract and converted into animal dose according to a dose conversion table.

All animal experiments in this study were approved by the Animal Experimentation Ethics Committee, The Chinese University of Hong Kong (CUHK4097/01M).

### Measurement of spinal BMD and body weight

Changes in bone mineral density (BMD) at rats' lumbar spine were monitored with pQCT (XCT2000, Stratec Medizintechnik, Germany) within the experimental period of 8 weeks. Quality assurance of measurements was checked with a hydroxyapatite cone and standard phantoms prior to the scanning of the rats each time. For the BMD measurement, the rats were first anesthetized with a cocktail of ketamine and xylazine (100 mg/kg body weight and 10 mg/kg body weight respectively) intramuscularly. They were then fixed on a custom-made translucent plastic holder to ensure achieving a repeatable positioning. The spines were scanned under the built-in research mode of the pQCT. Two slices were scanned at each site. The scan speed was 25 mm/sec with voxel resolution of 0.2 mm. The analytical parameters for trabecular BMD were set as threshold 280 mg/cm^3^, contour mode 1 and peel mode 20. The parameters for cortical BMD were set as threshold 551 mg/cm^3 ^and peel mode 2. The trabecular bone region was defined by setting an inner area to 35% of the total cross-sectional area. The body and uterus wet weights of all animals were monitored during the experimental period.

### Rat MSCs culture and differentiation

Rat MSCs were cultured from the bone of the tibiae and femora of the rats (250 g) by centrifugation as described previously [10]. Isolated bone marrow cells were resuspended in a growth medium consisting of α-MEM (Life Technologies, USA), 10% fetal bovine serum (Life Technologies, USA) and 1% penicillin/streptomycin (Life Technologies, USA), seeded at a density of 2 × 10^5^/cm^2^, and incubated at 37°C in 95% humidified air and 5% CO_2_. On day 7, all non-adherent cells were removed and followed by the medium change twice a week. The monolayer of adherent cells was trypsinized by 0.25% trypsin EDTA when it reached half-confluent and re-seeded at a density of 1 × 10^4^/cm^2 ^(passage 1, P1). Passage 2 (P2) culture was used for all *in vitro *assays. The identity of the culture was characterized by flow cytometry with CANTO ll using FACs Diva software (Version 5, BD Biosciences, USA), which showed positive results in two MSCs cell-surface molecules CD44 and CD90 and negative results in two hematopoietic markers, CD11b and CD54.

For the differentiation studies, P2 MSCs were seeded in the six-well plates of 2 × 10^4^/cm^2^. After three days, the growth medium was replaced with osteogenic medium (growth medium supplemented with 100 nM dexamethasone, 50 μg/ml ascorbate-2-phosphate and 10 mM β-glycerol phosphate) or adipogenic medium (growth medium supplemented with 1 μM dexamethasone, 50 μg/ml insulin, 0.5 mM methyl-isobutylxanthine and 100 μM indomethacin), with medium changed twice a week. FLL was prepared as stock solution in PBS and sterilized by filtration with 0.22 μm filter. This was then added to both differentiation media to provide final concentrations in the range within 100 μg/ml. Genistein and troglitazone at 20 μM was used as positive control in both differentiation protocols as reported [11,12].

### Cell viability assay

The cell viability of undifferentiated MSCs was determined by the 3-[4,5-dimethylthiazol-2-yl]- 2,5-diphenyl-tetrazolium bromide (MTT; Sigma, USA) assay after 48 hours of treatment with FLL aqueous extract (Sigma, USA) at various concentrations in 96-well plates (5×10^2 ^cells/well). The relative amount of viable cells was determined by measuring the reduction of MTT dye in live cells to blue formazan crystals at optical density at 540 nm and expressed as the percentage of control group without FLL treatment.

### Assessment of osteogenic and adipogenic differentiation markers

To determine the effect of FLL aqueous extract on osteogenesis in MSCs, we measured the related biochemical markers, namely alkaline phosphatase (ALP) activity and matrix calcium deposition. ALP activity was measured in the cell culture with the commercially available ALP assay kit (Stanbio, USA) after seven and 14 days of osteogenic treatment. Total protein content was determined with BCA protein assay reagent (Sigma, USA) and enzyme activities were expressed as U/mg protein. The matrix calcium deposition was quantified with StanbioTotal LiquiColor calcium determination kit (Stanbio, USA) after 14 days of osteogenic treatment and the readout was normalized by protein content determined by BCA assay reagent (Sigma, USA). The calcification was visualized by staining the cells with 2% Alizarin Red S solution for five minutes after the fixation with 10% buffered formalin (v/v) for 30 minutes. For adipogenesis, the number of adipocytes was determined with the Oil Red O staining method [11]. After 21 days of FLL-treatment in adipogenic medium, the culture cells were rinsed twice with PBS and fixed with 10% buffered formalin (v/v) for 10 minutes. Fixed cells were washed and stained with 0.2% Oil Red O-isopropanol for 15 minutes. Excessive stain was removed by distilled water for three times. Photomicrographs were taken with inverted microscope at 100× magnification. The number of adipocytes was calculated by counting the Oil Red O positive cells in 16 separated fields.

### Assessment of OPG and RANKL gene expressions on FLL-treated osteogenic culture

Total RNA was isolated from cultures of each experiment group with RNeasy mini kit (Qiagen, USA) on day 14. Total RNA was subjected to one-step real-time reverse transcription PCR using the QuantiFast SYBR Green RT-PCR kit (Qiagen, USA) with ABI 7500 Fast Real-Time PCR System (Applied Biosystems, USA). The specific PCR primer sets were designed and tested by manufacturer (QuantiTect Primer Assays, Qiagen, USA) to detect osteoprotegerin (OPG, NM_012870) and receptor activator of nuclear factor-κB ligand (RANKL, NM_057149).

Glyceraldehyde-3-phosphate (GAPDH, NM_017008) was served as a housekeeping gene. Relative gene expression levels were presented as 2^(-ΔCt) ^where ΔCt = Ct_target _- Ct_GAPDH _and the Ct was the cycle threshold. Standard deviation (SD) was determined from three independent experiments of the ΔCt values. The upper and lower errors were defined as 2^-(ΔCt-SD) ^and 2^-(ΔCt+SD) ^respectively. Fold change for the treatment group was defined as the relative expression, compared with the control group without treatment and was calculated as 2^(-ΔΔCt) ^where ΔΔCt_treatment _= ΔCt_treatment _- ΔCt_control_.

### Statistical analysis

Differences between treatment and control groups (not exposed to FLL extracts) were tested by one-way ANOVA, followed by *post hoc *Dunn's test. All statistical analyses were performed with the Statistical Package of Social Science (SPSS) version 15.0 (SPSS, USA). All statistical tests were carried out at the 5% level of significance (*P *< 0.05). Data were expressed as mean ± standard derivation (SD).

## Results

### FLL protects the lumbar spine from BMD loss without affecting body weight

**Figure 1:**
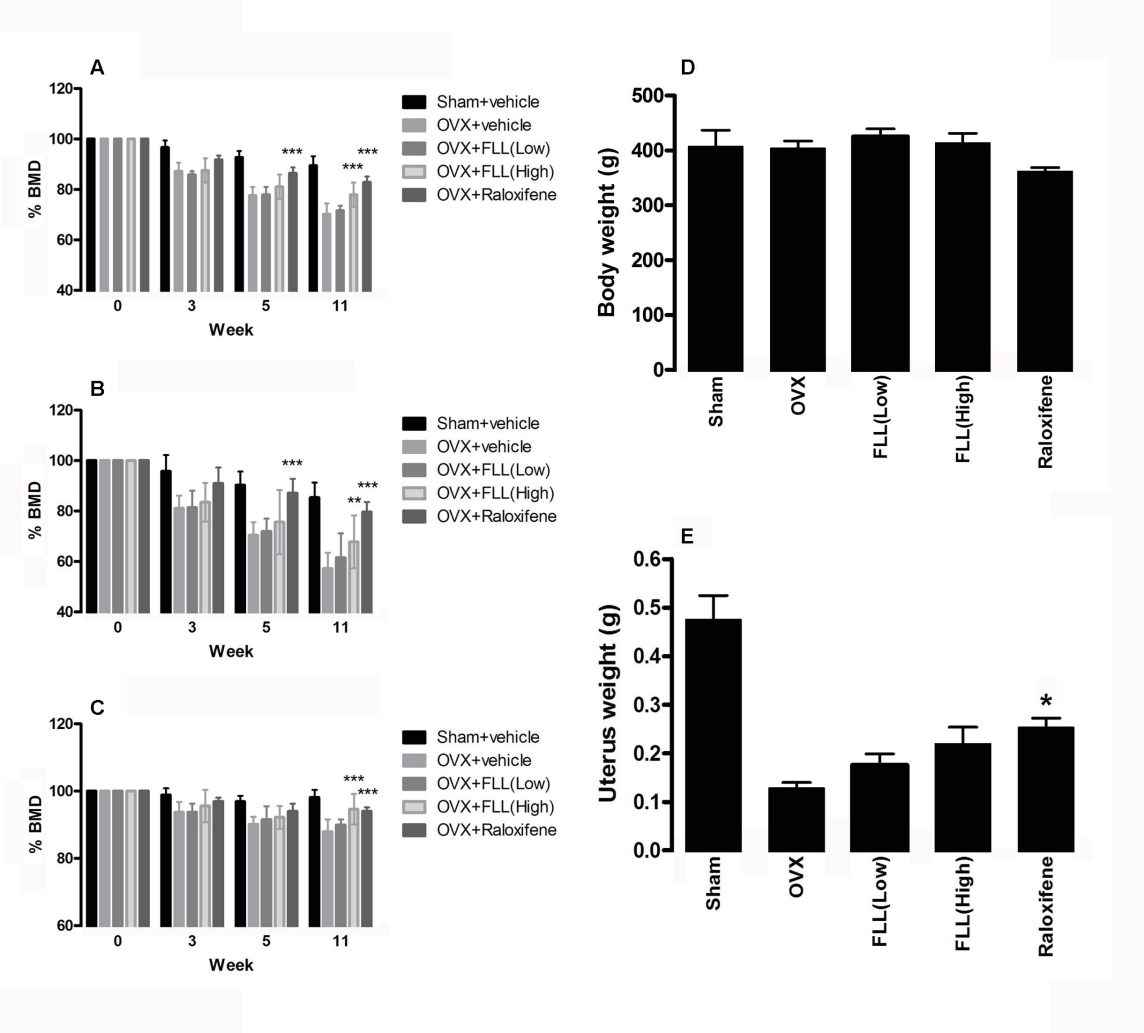
**Mean of percentage difference of BMD compared with week 0 in lumbar spine between the baseline (week 3, before ovariectomized), week 0 (ovariectomized) and week 8 after treatments**. (A) Total BMD; (B) trabecular BMD; (C) cortical BMD of lumbar spine were illustrated. The error bar represents the SD. Significant difference: * *P *< 0.05; ** *P *< 0.01; *** *P *< 0.001 for difference from OVX group without treatment. (Note: all groups were started from 100% at week 3.) (D) The body and (E) the uterus wet weight of different treatment groups were measured at the end of experiment.

BMD change of total, trabecular and cortical regions were illustrated in Figure 1(A-C) respectively. In lumbar spine, an insignificant decrease of BMD was observed in sham-operated group from week 3 to week 8 in both total and trabecular regions. This decrease was possibly due to the low mineral diet the animals following surgery. No obvious change was found at cortical region. For OVX group, significant reductions of BMD were detected in all studied regions over the experimental period after operation. At week 8, the BMD of total, trabecular and cortical regions were notably decreased by 29.8%, 42.8% and 12.0% respectively (*P *< 0.001). By contrast, the administration of high dose of FLL aqueous extract (0.7 g/kg/day) significantly reduced the BMD loss in the OVX group. At week 8, the BMD loss was greatly improved by 7.7% (*P *< 0.001), 10.6% (*P *= 0.004) and 6.7% (*P *< 0.001) in total, trabecular and cortical regions respectively, when compared with the vehicle-treated OVX group. Its efficacy was similar to the standard anti-osteoporotic drug, namely raloxifene (2.5 mg/kg/day). However, no significant improvement was found in the group treated with lower dose of FLL extract. For the body weight analysis, there was no significant difference between all treatment groups and the untreated OVX group (Figure 1D), indicating that FLL aqueous extract did not cause any significant side effect *in vivo *after 8 weeks of treatment. Ovariectomy brought about a decrease of 73% of the uterus weight (*P *< 0.001) (Figure 1E). Such loss was less pronounced when OVX rats were given FLL at high dose (*P *> 0.05 vs OVX) or raloxifene (*P *= 0.039 vs OVX) although it was still about 50% lower than that in the sham rats group (*P*< 0.001).

### Characterization of rat MSCs

**Figure 2:**
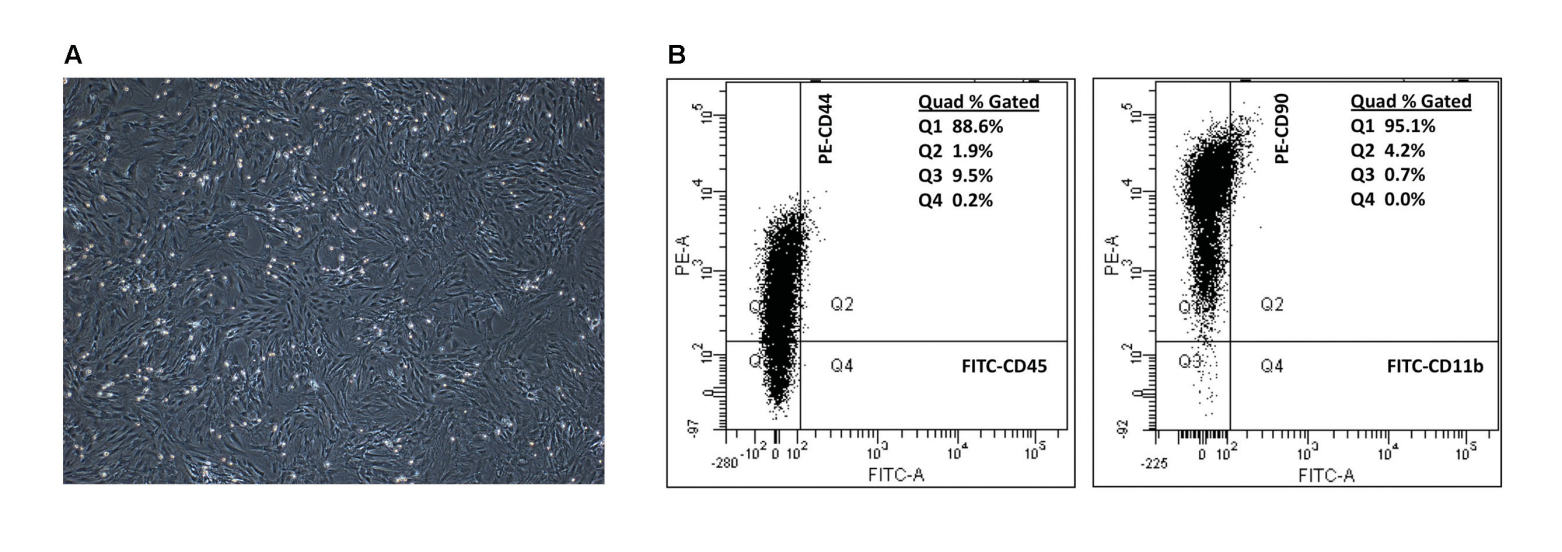
**Morphology and characteristics of bone marrow derived rat MSCs at passage 2**. (A) Rat MSCs maintained homogenous fibroblast-like appearance after adherent culture. (B) Cell surface markers were assessed using flow cytometry. Fluorescence intensity dotplots with specific antibodies (Ab); Phenotypes of MSCs labeled with Ab against PE-CD44 (with FITC-CD45 negative; left) and PE-CD90 (with FITC-CD11b negative; right). All experiments were performed in triplicate.

Rat MSCs were isolated from primary bone marrow cells and a homogeneous fibroblastic layer was formed (Figure 2A). As there is no single specific cell marker for MSCs, a panel of markers for flow cytometry was chosen. P2 rat MSCs culture was consistently expressed for CD44 (homing-associate cell adhesion molecules) and CD90 (Thy-1) but was negative for CD11b (monocyte/macrophage lineage marker) and CD45 (leukocyte common antigen) (Figure 2B).

### Effect of FLL on cell viability

**Figure 3:**
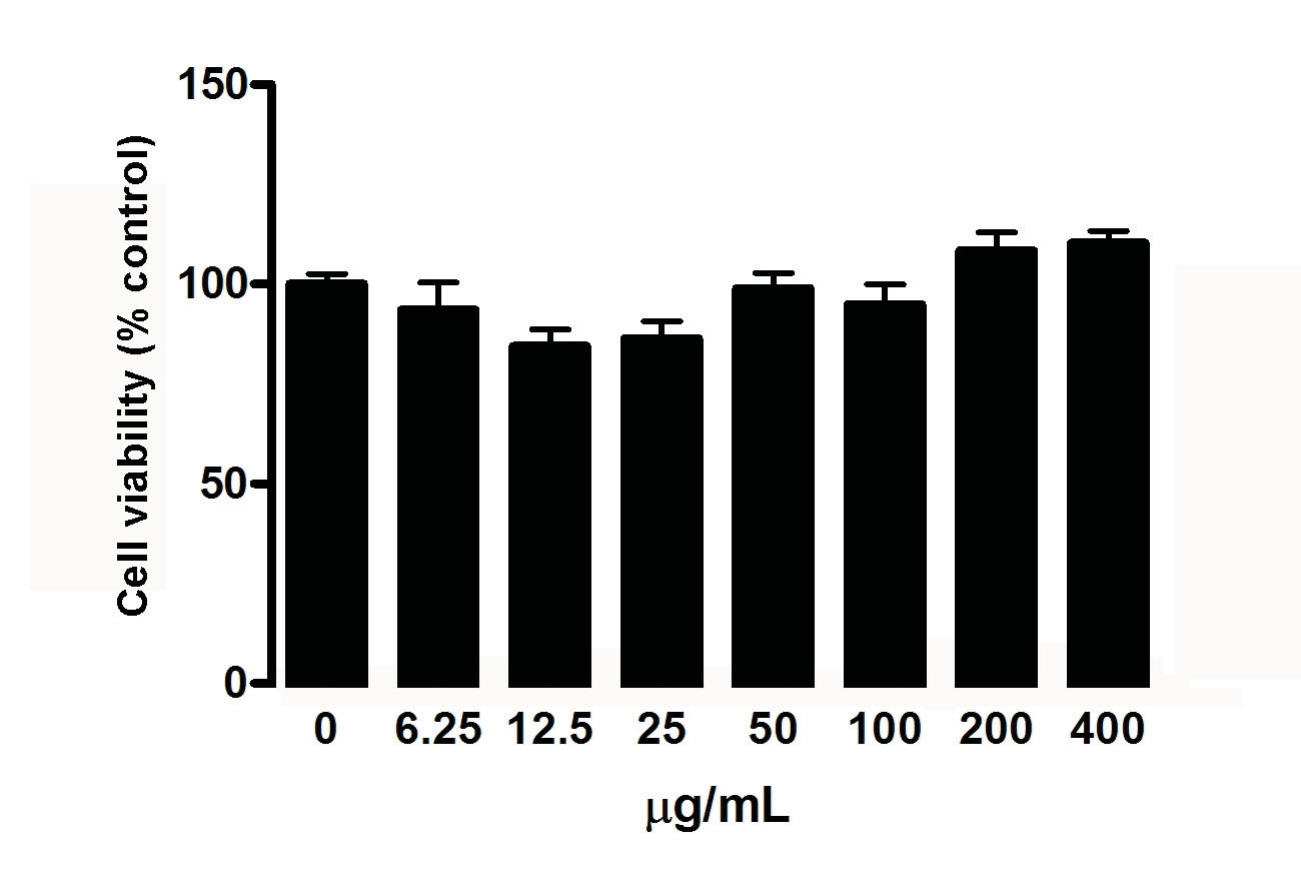
**Effect of FLL aqueous extract on the cell viability of P2 undifferentiated rat MSCs**. MSCs were treated with different concentrations of FLL extract for two days. The cell viability was estimated by MTT assay and the results are expressed as mean ± SD of three independent experiments each in triplicates.

There was no cytotoxic effect of FLL within a range of 0-400 μg/ml FLL extract on undifferentiated MSCs (Figure 3).

### FLL enhances osteogenic differentiation in MSCs

**Figure 4:**
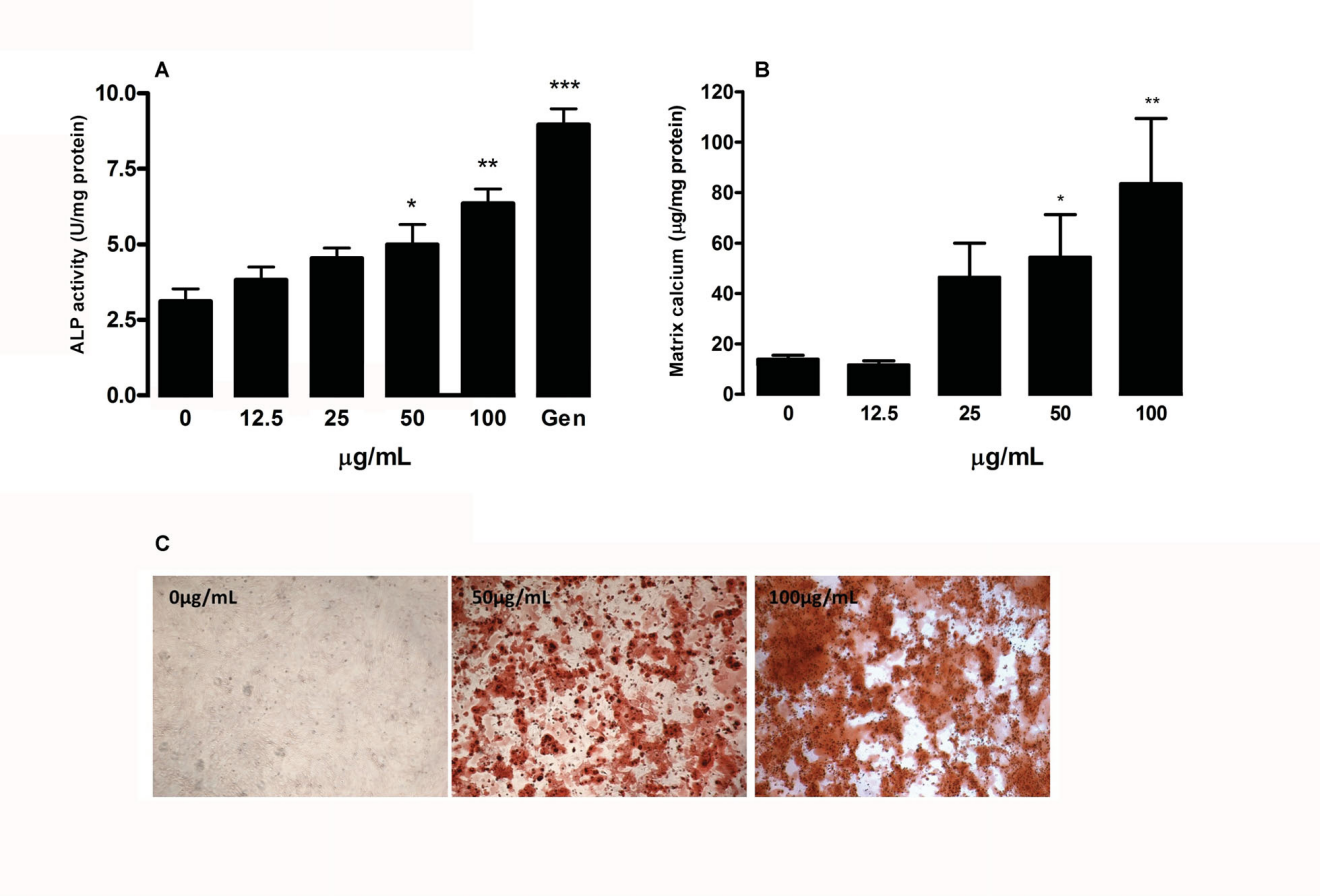
**Osteogenic properties of FLL aqueous extract in rat MSCs**. Dose effect of FLL aqueous extract on (A) alkaline phosphatase (ALP) activity and (B) extracellular matrix content in MSCs were determined under osteogenic induction on day 7 and day 14 respectively. Data are the mean ± SD (*n *= 3) from three independent experiments. Significant difference: * *P *< 0.05; ** *P *< 0.01; *** *P *< 0.001 for difference from respective baseline cultures without treatment. (C) Calcium deposition at different treatment concentrations (0, 50 and 100 μg/mL) was visualized by Alizarin Red S staining on day 14.

**Figure 5:**
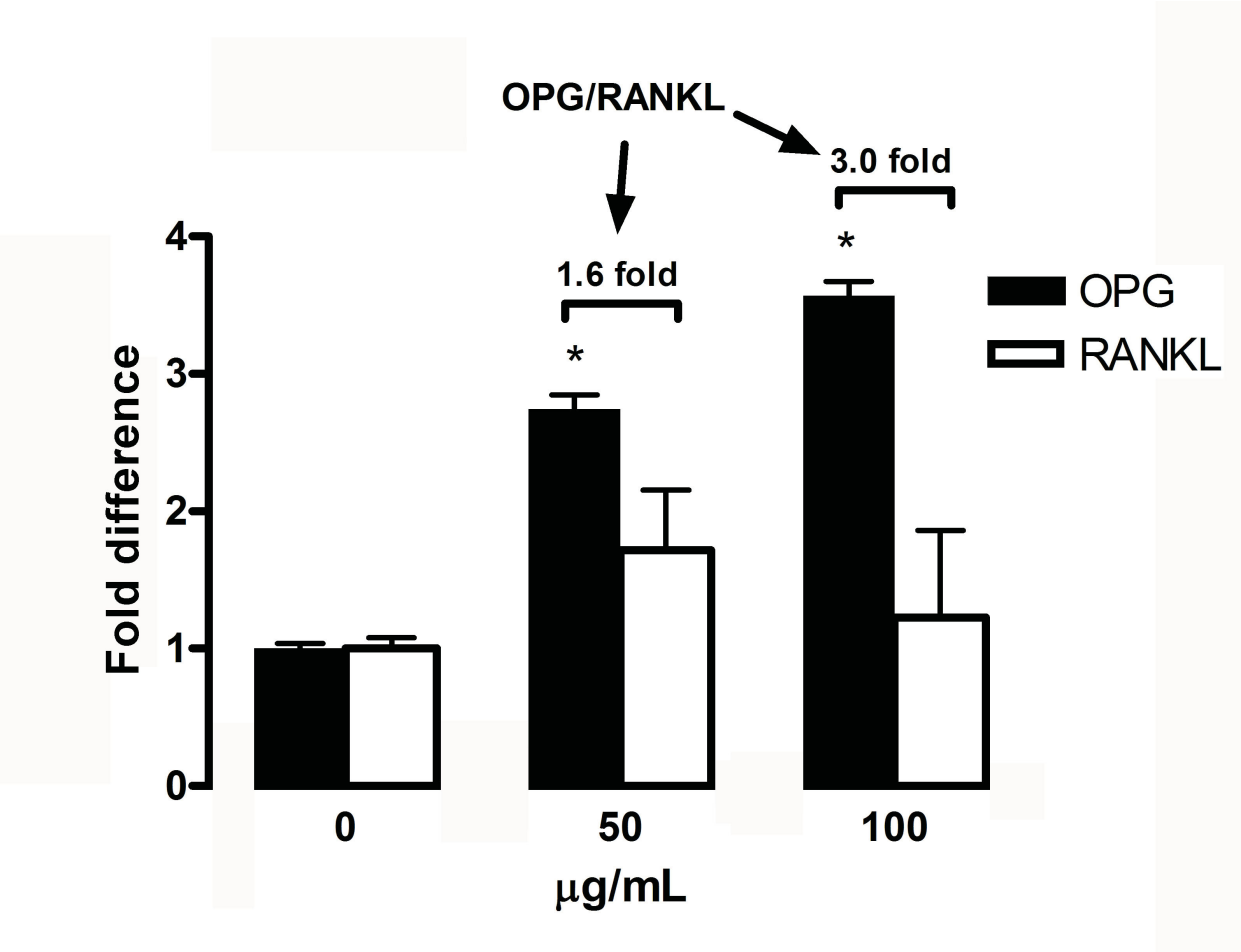
**Effect of FLL aqueous extract on mRNA expression of OPG and RANKL compared with control in rat MSCs under osteogenic induction on Day 14**. The mRNA expression levels of OPG and RANKL were determined using real-time PCR. The expression ratio of OPG-to-RANKL was also calculated. The expression levels of both genes were normalized on the basis of GAPDH expression. Fold difference were determined as the relative expression, compared with control cells without FLL treatment. Data are the mean ± SD (*n *= 3) from three independent experiments. * *P *< 0.05 for difference in OPG mRNA expression from culture without FLL treatment.

To investigate whether FLL aqueous extract affects osteogenic differentiation in MSCs, we determined the ALP activities in MSCs treated with different concentrations of FLL in the range without cytotoxicity (0-100 μg/ml). As shown in Figure 4A, ALP activities were increased in a dose-dependent manner (50-100 μg/ml, *P *= 0.004 to 0.032) after seven days of induction, compared with the respective control without treatment. In parallel, comparable increase of ALP activities was observed with the positive control genistein on day 7 by 33%. Effect of FLL on osteogenic differentiation as evidence by extracellular matrix calcium mineralization was also investigated (Figure 4B and Figure 4C). At 100 μg/ml, FLL significantly increased the matrix calcium deposition by about 6.4 folds (*P *= 0.003). Dose dependent response of calcium deposition was observed at lower concentrations of FLL. In addition, the mRNA expressions of OPG and RANKL were determined with quantitative real-time PCR in the cultures treated in osteogenic medium with or without FLL. As shown in Figure 5 FLL treatment increases the gene expression of OPG thereby elevating the overall OPG-to-RANKL ratio in a dose-dependent manner from 1.6 to 3.0 fold(s). These results showed that FLL may inhibit the osteoclastogenesis, increase the relative portion of OPG expression and protect the bone from resorption.

### FLL inhibits adipogenic differentiation in MSCs

**Figure 6:**
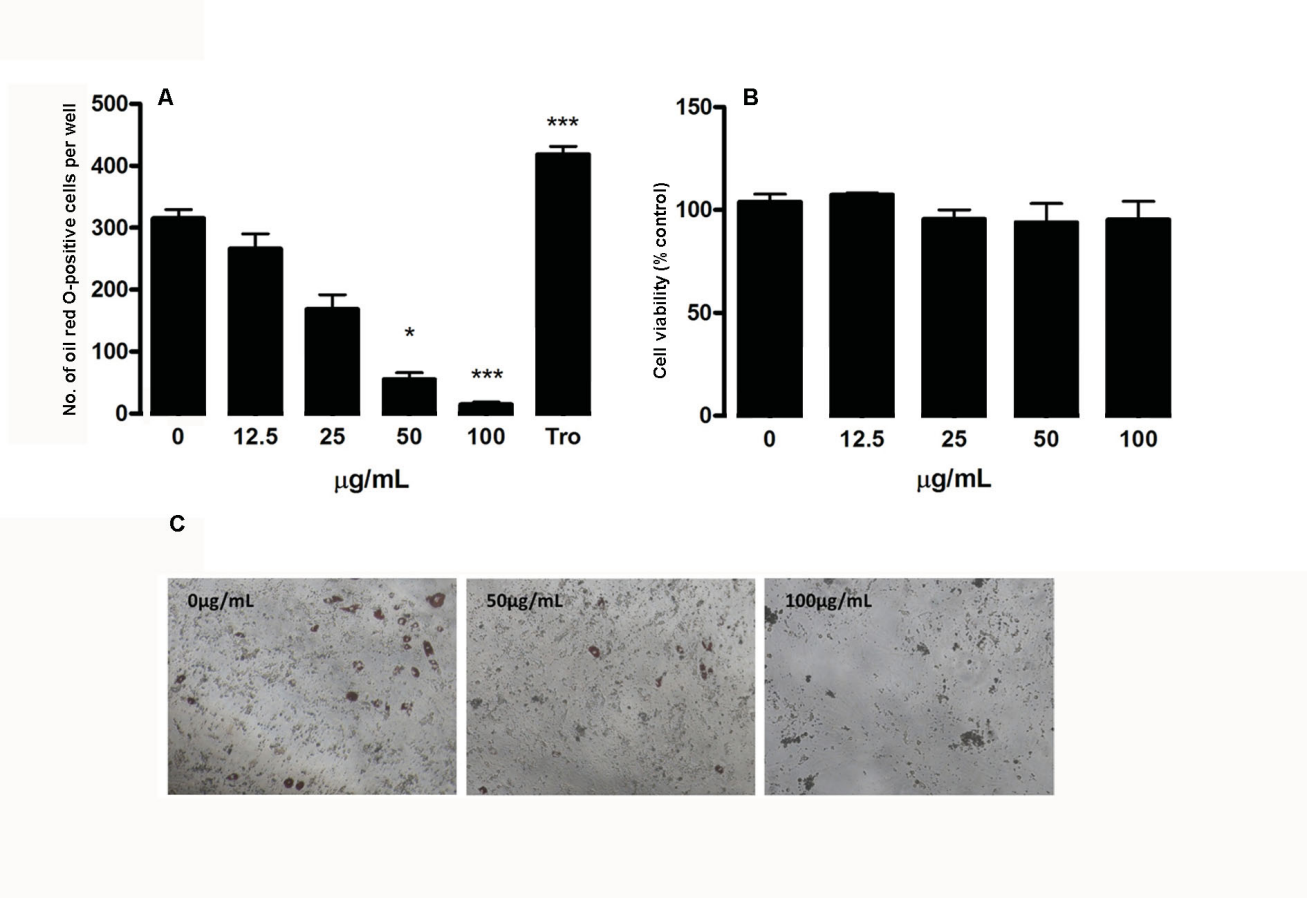
**Inhibitory effect of FLL aqueous extract on rat MSCs under adipogenic induction on day 21**. (A) The number of adipocytes was counted after Oil-red O staining. Troglitazone (Tro, 20 μM) acted as positive control. Data are the mean ± SD (*n *= 3) from three independent experiments. Significant difference: * *P *< 0.05; ** *P *< 0.01; *** *P *< 0.001 for difference from respective baseline cultures without treatment. (B) Effect of FLL aqueous extract on the cell viability of P2 adipogenic differentiated rat MSCs. MSCs were treated with different concentrations of FLL extract for 21 days. The cell viability was estimated by MTT assay and the results are expressed as mean ± SD of three independent experiments in triplicate. (C) Adipocyte deposition at different treatment concentrations (0, 50 and 100 μg/mL) was visualized by Oil Red O staining on day 21.

To investigate the effect of FLL aqueous extract on adipogenic differentiation in rat MSCs, we used Oil Red O staining to determine the degree of fat droplet accumulation in differentiated adipocytes after 21 days of adipogenic induction. As shown in Figure 6 FLL aqueous extract significantly inhibited the formation of adipocytes in a dose-dependent manner. At 50 μg/ml of FLL, the number of adipocyte was decreased by 82.4%. Similar response to 100 μg/ml of FLL was also observed, which inhibited the adipogenesis in MSCs by 95.3%. By contrast, the positive control troglitazone on day 21 increased the number of adipocyte by 33%. As shown in Figure 3B, our data demonstrated that that FLL did not affect the cell viability in a range of 0-100 μg/mL FLL on day 21, suggesting that this extract inhibited adipogenesis without affecting cell number.

## Discussion

The present study demonstrates that the FLL aqueous extract could protect the bone in standardized, aged-OVX rats via oral administration. In particular, oral administration of FLL dose-dependently reduced the lumbar spine bone mineral density loss in aged-OVX rats. FLL significantly improved the total, cortical and trabecular BMD in lumbar spine. The improvement was similar to that in the positive control raloxifene at week 8. Our in-house pilot study showed that the aqueous extract of FLL only slightly decreased the BMD loss in tibia by 5.0%, 4.9% and 1.0% in total, trabecular and cortical regions respectively, compared with the respective control group without treatment (*P *> 0.05; data not shown). Moreover, treatment with FLL at high dose was shown to have slightly mitigated the post-OVX deleterious effect on uterus without significant hypertrophic effects. Furthermore, FLL treatment did not decrease the body weight, suggesting that FLL exerted beneficial effects on bone without inducing potential side effects *in vivo*. FLL stimulated bone formation in osteoprogenitor rat MSCs without cytotoxic effects as indicated by the elevation of ALP activity and extracellular matrix mineralization. FLL significantly increased the OPG-to-RANKL (OPG/RANKL) ratio in MSCs thereby inhibiting osteoporosis-related osteoclast formation. Despite these pro-osteogenic effects in rat MSCs, FLL inhibited adipogenesis as indicated by the decrease of adipocytes numbers.

Our results are consistent with the literature on pro-bone actions of FLL ethanol extract in UMR-106 [4] and human mesenchymal stem cells [13]. Similarly, our data demonstrated that FLL aqueous extract increased extracellular calcium deposition on day 14, probably through the induction of early differentiation marker ALP activity upon treatment, suggesting that the composition of aqueous and ethanol extract may share some similarities. Nonetheless, aqueous extract is commonly used in Chinese medicine. The present study provided the first demonstration of the biological effects of FLL aqueous extract in adipogenic inhibition.

RANKL is essential in the maturation and activity of bone resorpting osteoclasts while OPG is a decoy receptor inhibiting osteoclast differentiation through its binding to RANKL [14]. A decrease in the OPG/RANKL ratio favors the osteoclastic activities and leads to the development of skeletal abnormalities [15]. Recently, estrogen has been reported to be an up-regulator of OPG in human [16] and clinically used for postmenopausal osteoporosis [17]. Our data showed that FLL aqueous extract significantly increased the OPG/RANKL ratio, suggesting that FLL protected the bone from osteoclastic resorption by modulating the OPG/RANKL system in rat MSCs.

Most of the osteoprotective agents have potential adverse effects. For example, estrogen replacement therapy is associated with increased risk of cancer [18] and cardiovascular diseases [19]. Anti-resorptive bisphosphonate therapy may lead to complications on upper gastrointestinal tract [20] and long-term effects on the skeleton, particular with respect to bone turnover and strength are unclear [21]. Our results indicated that FLL aqueous extract was non-toxic to aged OVX rat and MSCs. FLL or FLL-containing Chinese medicine formulae had relatively low toxicity *in vivo *[9,22].

pQCT, measuring the true volumetric BMD which is not size dependent, allows separate measures of BMD of the trabecular and cortical bone compartments. This technique provides geometric and structural parameters of bones which reflex skeletal strength [23]. Strong association was found between bone geometric parameters and failure loads in the spine and femoral neck [24,25], which suggested that pQCT and bone properties were highly correlated. Although pQCT is highly correlated with the bone and since pQCT can only qualitatively reveal the biomechanical properties of the bone, for in-depth analysis, three-point bending test will be performed to evaluate the bone strength and anti-fracture capacity. Furthermore, micro-computed tomography experiment will be performed to assess the detailed micro-architecture of the bone.

As it is unclear about the active ingredients, biotransformation and pharmacokinetic properties of FLL aqueous extract, further studies should be conducted to isolate the active ingredients and delineate the molecular mechanism(s).

## Conclusion

The present study demonstrates the osteoprotective effects of FLL aqueous extract on aged OVX rats, stimulation of osteogenesis, inhibition of adipogenesis and osteoclastogenesis in MSCs.

## Abbreviations

FLL: *Fructus Ligustri Lucidi*; OVX: ovariectomized; pQCT: peripheral quantitative computed tomography; MSCs: mesenchymal stem cells; BMD: bone mineral density; OPG: osteoprotegerin; RANKL: receptor activator for nuclear factor-κB ligand; ALP: alkaline phosphatase

## Competing interests

The authors declare that they have no competing interests.

## Authors' contributions

CHK, CBSL, KPF and PCL designed the experiments. CHK, WSS and CPL conducted the experiments. All authors read and approved the final version of the manuscript.
